# Characterization of a heat resistant ß-glucosidase as a new reporter in cells and mice

**DOI:** 10.1186/1741-7007-8-89

**Published:** 2010-06-22

**Authors:** Susan C McCutcheon, Ken Jones, Sarah A Cumming, Richard Kemp, Heather Ireland-Zecchini, John C Saunders, Carol A Houghton, Louise A Howard, Douglas J Winton

**Affiliations:** 1 Cancer Research UK Cambridge Research Institute, Li Ka Shing Centre, Robinson Way, Cambridge CB2 0RE, UK; 2 Wellcome Trust Centre for Stem Cell Research, University of Cambridge, Tennis Court Road, Cambridge CB2 1QR, UK; 3 Division of Virology, University of Glasgow, Glasgow G11 5JR, UK; 4 Gene Targeting Facility, Babraham Institute, Babraham, Cambridge CB2 4AT, UK

## Abstract

****Background**:**

Reporter genes are widely used in biology and only a limited number are available. We present a new reporter gene for the localization of mammalian cells and transgenic tissues based on detection of the *bglA *(*SYNbglA*) gene of *Caldocellum saccharolyticum *that encodes a thermophilic β-glucosidase.

**Results:**

*SYNbglA *was generated by introducing codon substitutions to remove CpG motifs as these are associated with gene silencing in mammalian cells. *SYNbglA *expression can be localized *in situ *or detected quantitatively in colorimetric assays and can be co-localized with *E. coli *β-galactosidase. Further, we have generated a Cre-reporter mouse in which *SYNbglA *is expressed following recombination to demonstrate the general utility of *SYNbglA *for *in vivo *analyses. *SYNbglA *can be detected in tissue wholemounts and in frozen and wax embedded sections.

**Conclusions:**

*SYNbglA *will have general applicability to developmental and molecular studies *in vitro *and *in vivo*.

## Background

A fundamental technique in biological research is the use of reporter genes to track cells or tissues in developmental studies, to quantify or recognize gene expression from defined cis-regulatory elements and to normalise for differential uptake of DNA or delivery vectors in transfection experiments *in vitro *[1-4]. The most frequently used reporters are *Escherichia coli lacZ *gene encoding β-galactosidase (βgal), the green fluorescent protein (GFP) of *Aequorea victoria *and to a lesser degree human placental alkaline phosphatase [3,5,6]. In transgenic studies GFP tends to be the reporter of choice for studies at single cell or intracellular resolution or where viable cells need to be isolated by fluorescence activated flow sorting (FACS). Histochemical detection of *lacZ *is still widely used at the single cell/tissue level of resolution especially where visualization is in wholemounts of tissues or embryos. Here we present a new reporter protein for cellular and whole organism studies that is validated *in vitro *and by generating a Cre-reporter mouse in which the reporter is detected in histological sections following induction of Cre recombinase. This new reporter gene, termed *SYNbglA*, is based on the *bglA *gene (GenBank: Accession X12575), of the thermophilic bacterium *Caldocellum saccharolyticum *that encodes a β-glucosidase (βglu) thermostable to 85°C [7].

## Results

**Figure 1:**
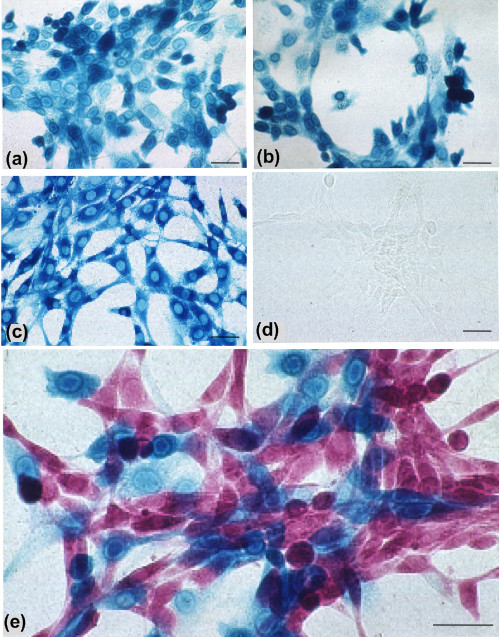
**Detection of β-glucosidase**. Clones of NIH 3T3 cells expressing *bglA *or *LacZ*: β-glucosidase (βglu) stained with BCI-glu (a, b); β-galactosidase (βgal)stained with BCI-gal (c, d); mixture of cells shown in a-d and stained with BCI-glu and Magenta-gal (e). Clones (b, d) received prior heat treatment (65°C, 20 min) showing that βglu (b) is still detectable histochemically while βgal (d) is not. In a mixed population, simultaneous incubation with both substrates results in cell autonomous staining for only one enzyme (e). Bars are 50 μm.

A mammalian expression construct in which the subcloned *C. saccharolyticum bglA *gene is regulated from the human elongation factor 1α (EF1α) promoter was generated to create pEF*bglA*. For comparison, *lacZ *was also subcloned to generate pEF*lacZ*. The EF1α promoter was chosen as, unlike powerful viral promoters such as the cytomegalovirus immediate early promoter (CMV IE1), it does not tend to undergo silencing over time [8]. Following transient transfection cells expressing thermostable βglu could be detected using BCI-glu in fixed cultures with or without heat-treatment (65°C for 20 min; data not shown). Colonies of NIH 3T3 cells transfected with pEF*bglA *and pEF*lacZ *were isolated. βglu expression remained detectable after heat treatment unlike βgal which was heat inactivated (Figure 1a-d). To establish if βgal and βglu can be co-localized stable clones separately expressing either reporter were derived and mixed in 1:1 ratio and cultured together for 1-2 days prior to fixation and staining with BCI-glu and Magenta-gal at 37°C. Individual cells in the co-cultures stained with one substrate only and showed no cross-reactivity demonstrating the potential to visualize these two reporters simultaneously (Figure 1e).

**Figure 2:**
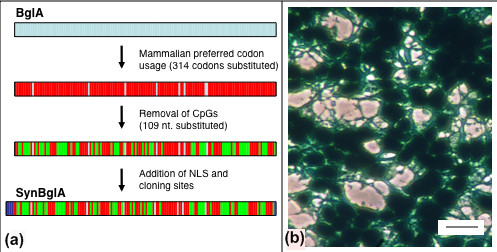
**Genesis and validation of *SYNbglA***. (a) Schematic of the nucleotide changes introduced to the *bglA *sequence to generate *SYNbglA*. Substitutions to introduce preferred mammalian codon usage (red infill) or remove CpG dinucleotides (green infill) are shown. 5'and 3'fusion elements (nuclear localization signal and cloning sites) are also shown (blue infill). (b) Detection of *SYNbglA*. BCI-glu stained clone of 293 cells obtained after transfection with CMV-*SYNbglA *showing intense nuclear localized and weaker cytoplasmic β-glucosidase up to at least passage 40. Bar is 50 μm.

After several passages, cultures derived from *bglA*+ clones in which all cells stained positive for βglu activity, exhibited large numbers of unstained cells. Such a 'silencing effect' has been reported in experiments with the E. coli *lacZ *gene [9]. Silencing of *lacZ *is ameliorated by changing its sequence to minimize the number of CpG dinucleotides that are targets for methylation in mammalian cells [9]. The *bglA *coding sequence contains 109 CpG dinucleotides. Consequently, we undertook to resynthesize *bglA *such that the nucleotide sequence was depleted for CpG dinucleotides and the codon sequence was biased towards mammalian usage. A nuclear localization signal was also added as a 5' fusion (Figure 2a). The reporter gene thus generated was termed *SYNbglA *(GenBank: Accession AY528410).

*SYNbglA *was subcloned to generate pCMV-*SYNbglA *that was cotransfected with pSV2*neo *into 293 cells and G418 resistant clones obtained. The CMV promoter was selected as a more robust test of the resistance of *SYNbglA *to silencing. Four clones were expanded and all expressed the altered βglu (SYNβglu) strongly and homogenously as evaluated by BCI-glu staining. The clones were serially passaged (cultures approaching confluency were split 1:4 to 1:8 every 2 or 3 days) and continuously evaluated for expression by staining with BCI-glu. No reduction in the proportion of cells stained or in their intensity was observed up to passage 40, the highest analysed (Figure 2b). We conclude that *SYNbglA *is not prone to the gene-silencing phenomenon observed with *bglA *and reported with unmodified *lacZ *[9,10].

**Figure 3:**
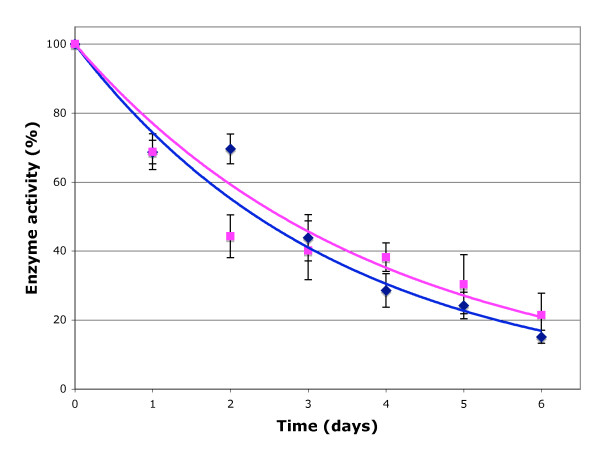
**Decay curves showing loss of reporter enzyme activity following removal of Pronesterone A from stable clones of 293EcR cells expressing SYNβ-glu (magenta square) and β-gal (blue diamond)**. Data was plotted as mean ± standard error of mean for SYNβglu (*n *= 4) and βgal (*n *= 2), where *n *= number of experiments per time point. Exponential decay curves were fitted, indicating half-lives of 2.65 days (*R*^2 ^= 0.913) and 2.33 days (*R*^2 ^= 0.969) for SYNβglu and βgal, respectively.

βgal expression can be quantified by detecting cleavage of *O*-nitro-phenyl-galactoside conjugated sugars in colorimetric assays. We confirmed that SYNβglu expression is quantifiable with ONP-glucopyranoside (not shown). This method was then used to gain insight into the functional stability of SYNβglu compared to βgal. Both proteins were expressed inducibly in stably derived clones of 293EcR cells (engineered to express a heterodimeric transcriptional transactivator that only binds to the DNA binding domain present in an inducible promoter in the presence of Pronesterone A, an ecdysone analogue) [11]. The rate at which reporter enzyme activity decayed following withdrawal of Pronesterone A was determined by harvesting treated cells daily for 6 days. Following the cessation of induced transcription, the rate of decay for SYNβglu is similar to that of βgal, with a 50% reduction in activity observed at 2.65 days and 2.33 days, respectively, suggesting that they have similar stabilities within cells (Figure 3).

**Figure 4:**
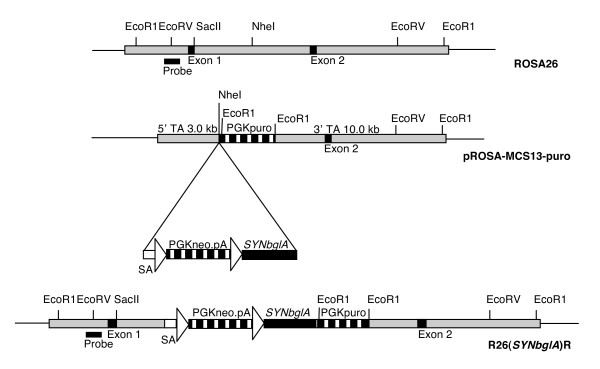
**Schematic of gene targeting at the ROSA26 locus to generate R26(*SYNbglA*)R**. Shown are: the ROSA26 locus (top); the targeting construct containing the loxP-PGK*neo*-loxP *SYNbglA *cassette (mid), arrows represent loxP sites; the targeted locus (bottom). Targeted clones were confirmed by Southern blotting of *Eco*R1 digested genomic DNA with the probe shown to generate 15 kb and 8.4 kb bands from the wild type and targeted alleles, respectively

**Figure 5:**
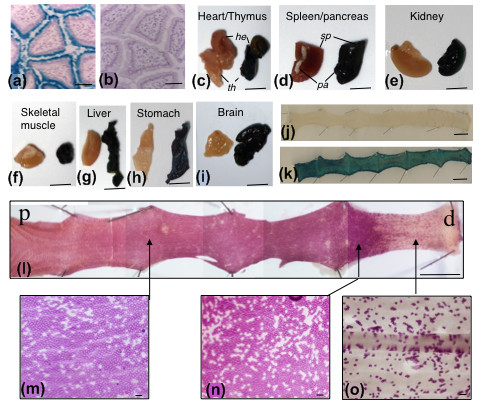
**Histochemical detection of β-glucosidase activity in mouse tissues**. (a, b) Cryostat sections of snap frozen small intestine from non-transgenic mice stained with BCI-glu. (a) Endogenous activity in the villus brush-border. (b) No activity following incubation at 65°C for 20 min. (c-i) BCI-glu staining of tissues from R26*SYNbglA*R (left of panel) and PGKcre^m^/R26*SYNbglA*R (right of panel) mice: (c) heart and thymus, (d) spleen and pancreas, (e) kidney, (f) skeletal muscle, (g) liver, (h) glandular stomach and (i) brain. (j, k) Small intestinal wholemounts heat treated at 65°C and stained with BCI-glu from: (j) untreated Ahcre/R26*SYNbglA*R; (k) β-napthoflavone treated Ahcre/R26*SYNbglA*R mice (4 weeks post induction), showing detectable enzyme activity only in (k). (l-o) Wholemount of colon from an Ahcre/R26*SYNbglA*R mouse prepared 16 weeks after β-napthoflavone treatment and stained with Mag-glu (p, proximal; d, distal). (l) Extensive recombination occurs throughout as indicated by magenta staining but becomes increasingly variegated towards the distal end. (m-o) Shows enlargements of areas indicated by arrows. Punctate magenta staining corresponds to individual colonic crypts. Bars: A, B 200 μm, C-O 1 cm.

In contemporary studies of gene function *in vivo*, genes are not only deleted or overexpressed constitutively but conditionally mutagenized using site-specific recombinases such as Cre [12,13]. Reporter lines for recombinase activity are necessary to determine the timing, spatial regulation and tissue specificity of recombinase expression [14]. Consequently, we decided to generate an alternative reporter line for assessing Cre activity by targeting the ubiquitously expressed murine ROSA26 locus with *SYNbglA *placed downstream of a floxed STOP cassette to create a line named R26(*SYNbglA*)R (Figure 4). Frozen sections and intestinal wholemounts from adult R26(*SYNbglA*)R and non transgenic animals were analysed for βglu activity using BCI-glu. Tissues from R26(*SYNbglA*)R and non transgenic animals showed identical staining patterns with all (including liver, oesophagus, bladder, pancreas, muscle and heart) except small intestine showing no staining due to background enzymatic activity. In the small intestine, in both cases, the positive staining was associated with the epithelial brushborder and could be prevented by incubation at 65°C for 20 min prior to incubation in BCI-glu (Figure 5a, b). These results indicate that there is no background expression from the targeted allele and that detectable endogenous βglu is only present in the small intestine.

In order to functionally test the R26(*SYNbglA*)R line it was mated to two different Cre expressing lines, PGKcre^m ^and Ahcre. PGKcre^m ^is expressed from a maternally inherited transgene that is expressed during the diploid phase of oogenesis resulting in complete recombination at loxP flanked cassettes even where PGKcre^m ^is not inherited [15]. Male R26(*SYNbglA*)R mice were crossed with PGKcre^m ^females and the offspring analysed around the time of weaning. Tissues were excised, fixed and stained as wholemounted tissues in BCI-glu for 48 h. Tissues from age-matched mice not crossed to PGKcre^m ^mice were also prepared. In all tissues of mice obtained from the R26(*SYNbglA*)R/PGKcre^m ^intercross, including heart, thymus, spleen, pancreas, kidney, skeletal muscle, liver, stomach and brain, there was intense staining with BCI-glu, where there was none in the control tissue. This demonstrates that SYNβglu expression is sustained in diverse tissue types following cre-mediated activation (Figure 5c-i).

Ahcre is a mouse line in which Cre recombinase is conditionally expressed from the rat cytochrome P450 IA1 promoter in several gastrointestinal tissues, following treatment with the inducing agent β-napthoflavone [16]. The Ahcre line was originally validated with the R26R reporter line, in which *lacZ *is expressed from the ROSA26 locus following cre-mediated recombination, allowing the known pattern of recombination to be compared with that obtained in Ahcre/R26(*SYNbglA*)R mice. Thus, Ahcre/R26(*SYNbglA*)R were either left untreated or treated with five daily intraperitoneal injections of 80 mg/kg β-napthoflavone to activate transcription of cre and mediate excision of the stop cassette allowing expression of *SYNbglA *(Figure 5j, k). There was very a very low level of background recombination in the target tissues of untreated adult animals (Figure 5j). After induction, there was near complete recombination with extensive expression of SYNβglu that could be detected with BCI-glu in small intestinal wholemounts (Figure 5k). In the colon of induced Ahcre/R26(*SYNbglA*)R mice there was extensive recombination that was maximal proximally and became increasingly mosaic towards the anus (Figure 5l-o). In both untreated and treated mice the pattern of recombination was identical to that observed previously in Ahcre/R26R mice although overall the extent of recombination as determined by expression of *SYNbglA *seems greater in more distal regions of the intestine [16].

**Figure 6:**
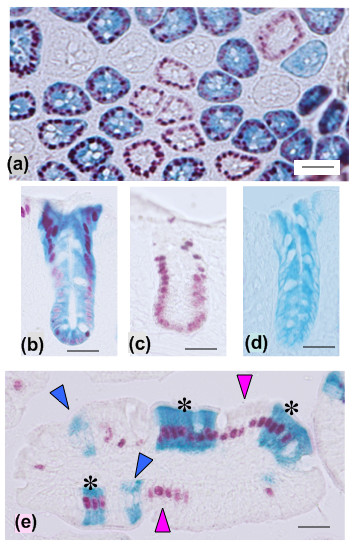
**Co-localization of βgal and SYNβglu in cryostat sections of intestine from Ahcre/R26R/R26(*SYNbglA*)R mice**. Colonic epithelium showing (a) transverse and (b-d) longitudinally cross cut crypts. (a) Shows a mixture of staining patterns with some crypts unstained (white), some being stained only with BCI-gal (blue, cytoplasmic) or Mag-glu (magenta, nuclear) and others staining for both. (b-d) Shows selected crypts staining for both BCI-gal and Mag-glu (b), Mag-glu only (c), BCI-gal only (d). (e) Showing cross cut small intestinal villus with several migration streams (originating from multiple crypts) present. Blue arrows, BCI-gal staining only; magenta arrows, Mag-glu staining only; asterices, combined BCI-gal and Mag-glu staining). Bars are 50 μm.

Increasingly transgenic experiments require the simultaneous application of two or more reporter genes. We wanted to establish if SYNβglu can be co-localized with βgal in tissues. In order to achieve this we chose to analyse the intestinal epithelium and exploit the known clonality of intestinal crypts [17]. Ahcre and reporter strains R26R and R26(*SYNbglA*)R were intercrossed to generate animals carrying all three modifications (Ahcre/R26R/R26(*SYNbglA*)R mice). These were injected with a single dose of β-napthoflavone to induce Cre submaximally such that mosaic patterns of recombination resulted. The intestines were then analysed for expression of both βgal and SYNβglu after 12 weeks, a time by which the process of crypt monoclonal conversion is largely complete [18]. Intestinal wholemounts or cryostat sections were stained first for βgal (6 h, 37°C) and then, after heat-treatment, for SYNβglu as described in the Methods section using BCI-gal and Mag-glu, respectively. Individual and clusters of stained crypts could be clearly identified and could be related to either reporter expressed alone or occasionally both together (Figure 6a-e).

**Figure 7:**
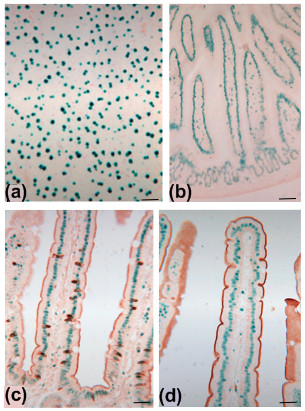
**Ahcre/R26*SYNbglA*R mice with tissues processed for paraffin wax embedding and stained with BCI-glu (37°C) for 48 h as described in the Methods section**. In liver (a) and small intestine (b) clear nuclear localized staining is evident. Small intestinal sections were further processed for immunohistochemistry with antibodies to muc2 (c) or villin (d) identifying goblet cells and absorptive enterocytes, respectively. Bars are 50 μm.

In order to determine if the introduced SYNβglu could be detected in sections processed for histology in paraffin wax blocks, different fixatives and protocols were tested. Liver, pancreas, bladder and small intestinal samples from Ahcre/R26(*SYNbglA*)R mice induced with β-napthoflavone 1-6 weeks previously were fixed in various fixatives for 1-6 h, processed through an ascending series of ethanols, xylene and into paraffin wax at 65°C for embedding. Five micrometer sections were cut, dewaxed in xylene and rehydrated before incubation with BCI-glu at 37°C. Clear nuclear localized histochemical product was found in patterns identical to that observed with cryostat sections as described above (Figure 7a and 7b). The main determinant of staining intensity was the fixation protocol with the best results achieved after 1 h fixation with 2%formalin/0.2% glutaraldehyde.

In order to test whether or not SYNβglu can be localized with other markers, we performed immunohistochemistry specific for intestinal cell types in tissue sections already stained for BCI-glu (either for 2 days at 37°C or overnight at 65°C). Villus enterocytes were easily identified in such sections on the basis of their positive staining for villin as were intestinal goblet cells on the basis of staining for muc2 (Figure 7c and 7d).

## Discussion

The βglu encoded by *SYNbglA *is an easily detected and stable protein that seems an ideal cell marking reporter molecule. It has potential application to cell and molecular studies where gene expression has to be localized. Its potential applications *in vivo *include studies during development and in the adult where gene expression from defined promoter elements have to be detected or for the fate mapping of tissues or individual cells, for example in clonal studies [19,20].

Like βgal expression levels of SYNβglu can be quantified using colorimetric substrates. Such assays are routinely used for normalizing for vector uptake in transfection experiments with the stability of βgal making it suitable for this purpose. However, such colorimetric assays are relatively insensitive and, as has been pointed out previously for βgal, chemiluminescent substrates can greatly increase sensitivity [3]. Appropriate chemiluminescent substrates are available for the detection of SYNβglu but, to date, we have not attempted to apply them. However, the ultimate limit on sensitivity for βgal is the presence of background enzymatic activity from endogenous, mammalian β-galactosidases and it is likely that the ability to destroy such background by heat inactivation will mean that SYNβglu has an enhanced sensitivity over that of βgal [21].

SYNβglu may have advantages over *E. coli *βgal for cellular localization in some experimental settings. The thermostability of SYNβglu allows it to be visualized in high-resolution sections from paraffin wax embedded tissues. The size of the coding sequence for *SYNbglA *is 1.2 kb compared to 3.1 kb for *lacZ*. This smaller size is advantageous for viral delivery vectors where cloning of large inserts is problematic.

The similarity of SYNβglu and βgal both in terms of processing requirements for visualization and the relative stabilities of the two proteins, together with the observation that they can be can co-localized, suggests that they may be used in tandem in compound genetically modified transgenic animals where gene expression changes are being effected. The compatibility of SYNβglu and βgal for dual detection may become especially significant as transgenic analyses become more elaborate. For example, a stem cell marker gene (Lgr5) has been validated in mice by localizing stem cells using an enhanced yellow fluorescent protein targeted to the Lgr-5 locus along with a Tamoxifen activated Cre recombinase [22]. Recombination mediated by the latter is recognized in crosses to R26R mice as clones of cells expressing βgal. A recent study of neuronal function combines use of GFP and human alkaline phosphatase to allow simultaneous detection of cell bodies, neurites and presynaptic sites and envisages the potential to also detect βgal in crosses to mutant strains [23]. In this regard, *SYNbglA *will add to the limited platform of available reporters. The R26(*SYNbglA*)R mice described here will further permit detailed analysis of the pattern of Cre activity in mouse models in which recombination is restricted to specific cell types and tissues and to different stages of development.

## Conclusions

SYNβglu is an easily detected reporter protein that has a variety of applications *in vitro *and *in vivo *where cell tracking, accurate localisation and high sensitivity is required. SYNβglu may offer some advantages to the *E. coli *βgal but the ability to use these two reporter systems together suggests that they will complement each other and will be used in tandem. The Cre-reporter animals described here demonstrate the applicability of SYNβglu to transgenic tissues and in the analysis of Cre mediated recombination.

## Methods

### Plasmid cloning

The *bglA *sequence was initially PCR amplified from pNZ1065 (gift of Dr D Love) and subcloned into pCR3 (Invitrogen) using primers (5' ttc**catggGGATCC**taagtttcccaaaaggatttttgtgg 3' and 5' tt**AGATCT**gtcgacttacgaattttcctttatatactg 3') designed to introduce a consensus translation start sequence (bold) and flanking restriction sites (caps) for subsequent cloning [7,24]. This *bglA *fragment was subcloned into pEF1alpha [25] to generate an expression construct (pEF*bglA*) containing the human type 1α elongation factor promoter and SV40 polyadenylation sequence. In order to allow a comparison, the equivalent *lacZ *construct (pEF*lacZ*) was also made by conventional subcloning (cloning details available on request). The SYN*bglA *and *lacZ *cassettes were also subcloned into pIND (Invitrogen, CA, USA) containing five ecdysone response elements upstream of a Drosophila minimal promoter. Additional cloning details are available upon request.

In order to generate the final gene targeting construct, conventional cloning was performed to make a cassette comprising loxP-PGK*neo*.pA-loxP *SYNbglA*.pA which was subcloned into pROS-MCS-13 [26] containing the two arms of ROSA26 locus homology. Additional cloning details are available upon request.

### Cell lines

Mouse NIH 3T3 and human 293-EcR cells were obtained from the American Type Culture Collection and Invitrogen, respectively, and were maintained in standard tissue culture media (Dulbecco's Modified Eagle's Medium) containing 10% fetal calf serum. Cells were transfected using the Stratagene MBS kit. Where cotransfection was required for the purpose of antibiotic selection the test plasmid was cotransfected (10:1 ratio) with pSV2*neo *(Clontech, CA, USA) followed by G418 selection at 1 mg/mL.

### Synthesis of *SYNbglA*

*SYNbglA *was chemically synthesized by Bionexus using their proprietorial method (Bionexus Inc, CA, USA http://www.bionexus.net/)

### Gene targeting

Embryonic stem cell manipulation procedures were performed by the Gene Targeting Service, Babraham Institute (Cambridge, UK). E14 129Ola ES clones were selected with G418 and colonies initially screened by polymerase chain reaction using primers anchored 5' to the shorter targeting arm (R26TOPV: 5' ggtagtggggtcgactagatgaaggagagcc 3') and at the introduced splice acceptor (R26SAmut: gtcctcaaccgcgagctgtg) which amplified a unique 4kb band. Two clones were selected following further screening by Southern blotting using a probe located 5' to the targeting vector as described previously [26]. These clones, C8 and C10, were microinjected into blastocysts and the resultant chimeras used to establish R26(*SYNbglA*)R mice. Both clones were evaluated for expression of reporter following induction of Cre in double transgenic Ahcre/R26(*SYNbglA*)R mice and were found to behave identically.

### Enzyme histochemistry

Cellular localization for *lacZ *in tissue culture plates was performed by routine methods [27] that were modified for detection of *bglA *expression as needed. Essentially, plates were washed in phosphate buffered saline (PBS) before fixation in 2% formaldehyde/0.1% glutaraldehyde in PBS. After washing with PBS plates were incubated with 0.04% conjugated sugar substrates [5-bromo-4-chloro 3-indolyl-β-D-galactopyranoside (BCI-gal, No. MB1001), 5-bromo-6-chloro-3-indolyl-β-D-galactopyranoside (Magenta-gal, No. M1141), 5-bromo-4-chloro 3-indolyl-β-D-glucopyranoside (BCI-glu, No. MB1002), all from Melford Laboratories, Suffolk, UK or 5-bromo-6-chloro-3-indolyl-β-D-glucopyranoside (Magenta-glu, No. 31059, Glycosynth, Cheshire, UK)] containing potassium ferrocyanide (4 mM), potassium ferricyanide (2 mM) and magnesium chloride (1 mM) in PBS at the temperatures stated.

### Protein stability determination

The colorimetric assay for βgal and βglu was carried out using a commercially available kit (No. E2000; Promega, WI, USA) as per the manufacturers instructions, except that the substrate containing solutions [*o*-nitrophenyl-β-D-galactopyranoside (ONP-gal), Sigma No. N1127, *o*-nitrophenyl-β-D-glucopyranoside (ONP-glu), Sigma No. N8016] at a concentration of 1.33 mg/mL were prepared independently. The absorbance of the cleaved substrates at 420 nm was determined on a Tecan SpectraFluor Plus plate reader.

The commercially available ecdysone inducibility system was obtained including 293EcR cells that stably express the VgRXR receptor (a heterodimer of ecdysone receptor and retinoid X receptor) (Invitrogen, Scotland, UK). In 293 EcR cells the heterodimer binds to an ecdysone response element in pIND in the presence of Prontesterone A. Clones of 293EcR cells were obtained by selection with G418 following transfection with pIND*SYNbglA *or pIND*lacZ *that contain a neo selection cassette. Clones were screened for conditional expression of βglu and βgal, respectively, in the presence of Pronesterone A (5 μM). For each reporter one clone was plated out at low density in 2.5 cm^2 ^replicated tissue culture wells which were incubated in media containing Pronesterone A (5 μM) for 24 h. Triplicate wells for each clone were harvested in lysis buffer (Promega, No. 397A) at 24 h intervals after PBS wash (x3). Cells harvested at the end of Pronesterone A treatment were designated day 0. For each lysate total protein concentration was determined using a Pierce BCA kit (PerBio, No. 23227) and the volumes analysed in the enzyme assay were normalized for total protein content.

### Treatment of animals

Homozygous Ahcre mice were crossed with R26(*SYNbglA*)R animals and offspring carrying both transgenes selected for subsequent experiments. Ahcre mice were genotyped as described and R26(*SYNbglA*)R mice by polymerase chain reacton using a primer combination (5' cagaaaggtagacggatttagcc 3'; 5' gggatacagaagaccaatgcaga3'; 5' tcctcaaccgcgagctgtg 3') giving a 440 bp and 350 bp for the wild type and targeted R26 loci, respectively [16]. For induction of the Ah promoter, mice received interperitoneal injections of 80 mg/kg β-napthoflavone (βNF; Sigma) dissolved in corn oil (8 mg/mL) at the frequencies stated and controls received either no treatment or corn oil only.

### Tissues and immunohistochemistry

Whole tissues for BCI-glu staining were dissected from 3-week-old mice and were sliced to present a cut facet for histochemistry. Tissues were fixed in 4% paraformaldehyde for 2 h, washed in PBS and incubated in BCI-glu at 50°C for 48 h. Intestinal wholemounts from Ahcre/R26(*SYNbglA*)R animals were prepared lumenal side up as described previously [16] except that they were fixed in ice cold 2% formaldehyde/0.2% glutaraldehyde in PBS (pH7.4) for 1 hour prior to overnight incubation in BCI-glu (as described above) substrate at room temperature. For frozen sections small pieces of intestine were snap frozen in liquid nitrogen and cryostat sectioned. Slides were air dried, fixed for 5 minutes in ice cold 2% formaldehyde/0.2% glutaraldehyde in PBS (pH7.4) for 10 min, before transferral to BCI-glu. For heat inactivation sections were incubated in PBS at 65°C for 10-20 min prior to incubation in BCI-glu. Tissues processed for histology were immersed in the fixatives for the length of time stated and processed into paraffin wax blocks using a Citadel tissue processor (ThemoShandon, Cheshire, UK) with freshly prepared dehydrating ethanols (x1 70%, 30 min; x1 90%, 30 min; x3 100%, 30 min each), xylene (x3, 20 min each) and wax (x2, 30 min each). Sections were cut at 3-5 μm, dewaxed and rehydrated into PBS prior to immersion into BCI-glu, prepared as above, and incubated at 37°C (48 h) or 65°C (overnight).

## Abbreviations

BCI-gal: 5-bromo-4-chloro 3-indolyl-β-D-galactopyranoside; BCI-glu: 5-bromo-4-chloro 3-indolyl-β-D-glucopyranoside; βgal: β-galactosidase; βglu: β-glucosidase; βNF: β-napthoflavone; GFP: green fluorescent protein; Magenta-gal: 5-bromo-6-chloro-3-indolyl-β-D-galactopyranoside; Magenta-glu: 5-bromo-6-chloro-3-indolyl-β-D-glucopyranoside; PBS: phosphate buffered saline; SYNβglu: synthesized β-glucosidase

## Authors' contributions

SMC performed the experiments shown in Figures 1 and 2. KJ performed the experiments resulting in Figure 3. SAC performed first proof-of- principle transient transfections referred to in text. JCS generated the reporter mice. HI-Z performed the *in vivo *analysis. CAH performed the mouse genotyping. LAH performed the histology and histochemistry. RK performed the data analysis and participated in the experimental design and manuscript preparation. DJW conceived the study and participated in its design, coordination and drafting of the manuscript. All authors have read and approved the manuscript.
